# Application of the metagenomic next-generation sequencing technology to identify the causes of pleural effusion

**DOI:** 10.3389/fmed.2025.1525100

**Published:** 2025-03-19

**Authors:** Zhiyun Yan, Cheng Sun, Wanna Tang, Weitao Cao, Jin Lv, Zhike Liang, Shuquan Wei, Weinong Zhong, Ziwen Zhao, Zhuxiang Zhao, Yujun Li

**Affiliations:** ^1^The First Clinical Medical College, Guangdong Medical University, Zhanjiang, China; ^2^Department of Pulmonary and Critical Care Medicine, Guangzhou First People's Hospital, South China University of Technology, Guangzhou, China; ^3^Department of Pulmonary and Critical Care Medicine, Guangzhou First People's Hospital, Guangzhou Medical University, Guangzhou, China; ^4^The First Affiliated Hospital of Jinan University, Guangzhou, China; ^5^Radiology Department, Guangzhou First People's Hospital, South China University of Technology, Guangzhou, China

**Keywords:** metagenomic next-generation sequencing, pleural effusion, malignant pleural effusion, tuberculous pleural effusion, *Mycobacterium tuberculosis*

## Abstract

**Background:**

Pleural effusion (PE), frequently encountered in clinical practice, can arise from a variety of underlying conditions. Accurate differential diagnosis of PE is crucial, as treatment and prognosis are heavily dependent on the underlying etiology. However, diagnosing the cause of PE remains challenging, relying on mycobacteriological methods that lack sensitivity and are time-consuming, or on histological examinations that require invasive biopsies. The recent advancements in metagenomic next-generation sequencing (mNGS) have shown promising applications in the diagnosis of infectious diseases. Despite this, there is limited research on the utility of mNGS as a comprehensive diagnostic tool for simultaneously identifying the causes of PE, particularly in cases of tuberculosis or malignancy.

**Methods:**

This study aimed to assess the efficacy of mNGS in detecting tuberculous pleural effusion (TPE) and malignant pleural effusion (MPE). A total of 35 patients with PE were included, and their PE samples were analyzed using mNGS.

**Results:**

Among the participants, 8 were ultimately diagnosed with TPE, and 10 were diagnosed with MPE, with lung adenocarcinoma being the most prevalent pathological type (50%, 5/10), according to established diagnostic criteria. Additionally, 7 patients were diagnosed with non-infectious PE. However, mNGS identified only 2 cases of TPE and 8 cases of MPE. The sensitivity of mNGS for detecting *Mycobacterium tuberculosis* was 25% (2/8), while the specificity was 100%. For tumor detection, mNGS demonstrated a sensitivity of 80%, a specificity of 92.6%, and an AUC of 0.882.

**Conclusion:**

mNGS is effective in distinguishing MPE from non-MPE, but is not suitable for diagnosing TPE.

## 1 Background

Pleural effusion (PE), characterized by the abnormal accumulation of transudates or exudates within the pleural cavity, represents a common clinical manifestation of various pathological conditions. Epidemiological data indicate an annual incidence of approximately 1.5 million cases in the United States alone (1, 2). Based on Light’s criteria, pleural effusions are clinically classified into two distinct categories: transudative and exudative (1, 3). While the etiology of transudative PE is typically straightforward to identify, exudative PE present more complex diagnostic challenges, predominantly manifesting as either malignant pleural effusion (MPE) or tuberculous pleural effusion (TPE). This diagnostic complexity is particularly pronounced in China, where the high prevalence of tuberculosis (TB) significantly contributes to disease burden (4, 5). The clinical significance of MPE is particularly noteworthy, as it typically indicates advanced-stage malignancy and is associated with poor prognostic outcomes (5, 6). The critical need for early and accurate diagnosis of MPE is underscored by current diagnostic limitations. Conventional diagnostic approaches, including mycobacteriological examinations with inherent sensitivity limitations and prolonged processing times, or histological analyses requiring invasive biopsy procedures, remain suboptimal (7–9). These limitations highlight the pressing need for developing more sensitive and minimally invasive diagnostic modalities in clinical practice.

The advent of metagenomic Next-Generation Sequencing (mNGS) has revolutionized pathogen detection in clinical diagnostics, with applications extending across diverse biological specimens including plasma, bronchoalveolar lavage fluid (BALF), PE, and cerebrospinal fluid. This cutting-edge technology offers unparalleled advantages in clinical microbiology, particularly through its capacity to simultaneously identify uncultivable, novel, and unexpected pathogens in a hypothesis-free manner, independent of clinical presumptions (10, 11). Notably, mNGS analysis of respiratory specimens (sputum and/or BALF) has demonstrated superior sensitivity and diagnostic efficiency for pulmonary tuberculosis detection compared to conventional methods such as acid-fast bacillus (AFB) smear microscopy and mycobacterial culture (12). The clinical significance of PE, frequently associated with advanced malignancies including lung, breast, gastrointestinal, and ovarian cancers (13, 14), coupled with the characteristic genomic instability of neoplastic cells (15), presents a unique diagnostic opportunity. Recent advancements have expanded the application of mNGS beyond pathogen detection, enabling the identification of malignant cells through analysis of genomic instability patterns (16). Emerging evidence suggests that mNGS analysis of body fluids can facilitate the detection of occult malignancies via copy number variation (CNV) (16), while innovative pipelines have been developed for simultaneous pathogen and cancer detection using Illumina sequencing of lung biopsy specimens (17). Despite these technological breakthroughs, the potential of mNGS as a comprehensive diagnostic tool for differentiating between malignant and infectious (particularly tuberculous) etiologies of PE remains to be fully elucidated.

In this study, we conducted a comprehensive investigation to evaluate the diagnostic potential of mNGS in PE analysis. A cohort of 35 patients with clinically confirmed PE was prospectively enrolled, and their pleural fluid samples were subjected to mNGS analysis to assess its diagnostic performance and clinical utility.

## 2 Methods

### 2.1 Patients and study design

This study was conducted at Guangzhou First People’s Hospital from March 2022 to February 2023, enrolling 40 patients with radiologically confirmed PE through computed tomography (CT) and/or ultrasonography. The inclusion criteria required definitive PE diagnosis via imaging modalities, while exclusion criteria comprised: (1) age < 18 years; (2) pregnancy or lactation women; (3) contraindications to thoracentesis procedures; (4) hypersensitivity to local anesthetics; (5) coagulopathy (INR >1.5 or platelet count <50 × 10^9^/L); (6) localized infection at the puncture site; (7) patient declination of participation; and (8) failure to meet predefined specimen quality control standards (detailed in the “NGS Sequencing” section). After rigorous screening, 35 participants were included for final analysis.

Standardized data collection protocols were implemented to systematically document demographic profiles, clinical laboratory parameters (including biochemical and cytological analyses), radiographic findings, and comprehensive medical histories. All participants underwent parallel diagnostic testing with acid-fast bacilli (AFB) staining, conventional microbial culture, mNGS, and histopathological evaluation.

**Figure 1:**
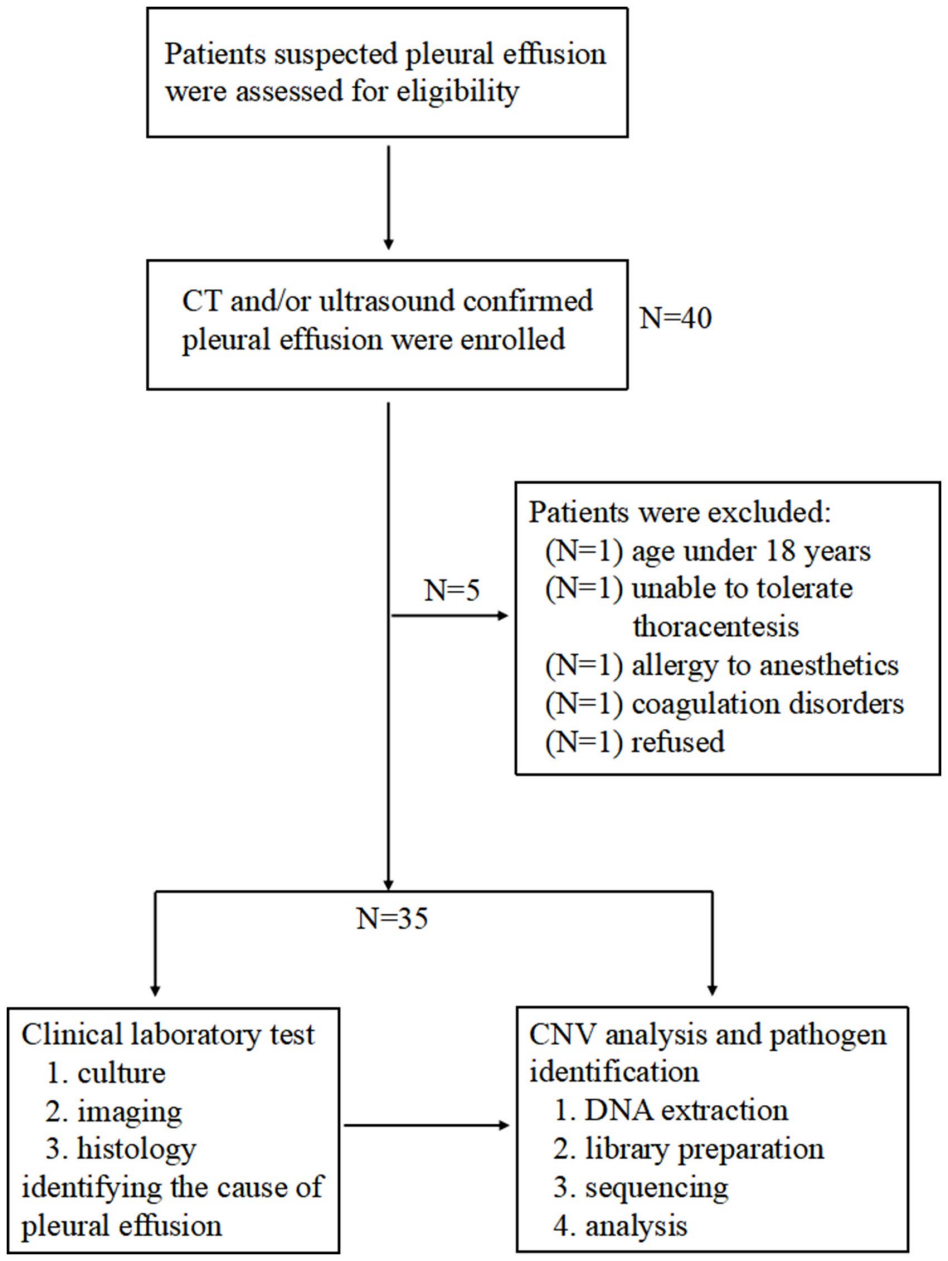
Study flowchart.

The study protocol received ethical approval from the Institutional Review Board of Guangzhou First People’s Hospital (Approval ID: K-2022-113-01). Written informed consent was obtained from all participants prior to sample collection. Pleural fluid specimens were processed at Hangzhou Jieyi Biotechnology Co., a CAP-accredited clinical genomics laboratory, adhering to standardized protocols endorsed by the hospital’s ethics committee. A schematic representation of the experimental workflow is provided in Figure 1.

### 2.2 TB detection

The diagnostic workflow for TB detection incorporated standardized microbiological analyses performed on PE, sputum, and/or BALF specimens. AFB staining was conducted using Ziehl–Neelsen staining kits (BASO Diagnostics, Zhuhai, China), while mycobacterial culture utilized Roche Middlebrook 7H11 agar slants (Kailin Trading Co. Ltd., Jiangmen, China), with all procedures adhering to standardized clinical protocols and manufacturer specifications.

Diagnostic confirmation of tuberculous pleuritis (TBP) required fulfillment of at least one of the following gold-standard criteria: (1) microbiological confirmation through AFB smear positivity and/or *Mycobacterium tuberculosis* culture isolation, or (2) histopathological identification of caseating granulomas in pleural tissue biopsies, as established in current clinical guidelines (16, 17).

### 2.3 Non-TB infection

The diagnosis of non-tuberculous infections was established through a composite of laboratory and clinical criteria. Patients were classified as non-TB infections if they fulfilled at least one of the following: (1) microbiological confirmation via positive conventional microbial culture or mNGS identifying non-tuberculous pathogens; (2) clinical diagnosis supported by characteristic manifestations (e.g., nocturnal paroxysmal dyspnea) and objective therapeutic response to non-tuberculous antimicrobial therapy during follow-up evaluations.

### 2.4 Malignant tumor identification

The diagnosis of MPE was histopathologically confirmed through microscopic examination of either pleural tissue biopsies or cytological preparations from PE sediment. All specimens were processed using standard hematoxylin and eosin (H&E) staining protocols. In diagnostically challenging cases, immunohistochemical (IHC) analyses were performed to establish definitive pathological classification and identify specific tumor subtypes, following established diagnostic guidelines.

### 2.5 NGS sequencing

PE samples were processed for mNGS following established protocols (18, 19), Genomic DNA was extracted using the Nucleic Acid Extraction Kit (Cat. MD013, MatriDx Biotech Corp., Hangzhou, China) and subsequently prepared for sequencing with the Total DNA Library Preparation Kit (Cat. MD001T, MatriDx Biotech Corp.) on an NGS Automatic Library Preparation System (Cat. MD005, MatriDx Biotech Corp.). Prepared libraries were pooled and sequenced on an Illumina NextSeq500 platform (Illumina, San Diego, CA, USA) using a 75-cycle high-output sequencing kit.

**Figure 2:**
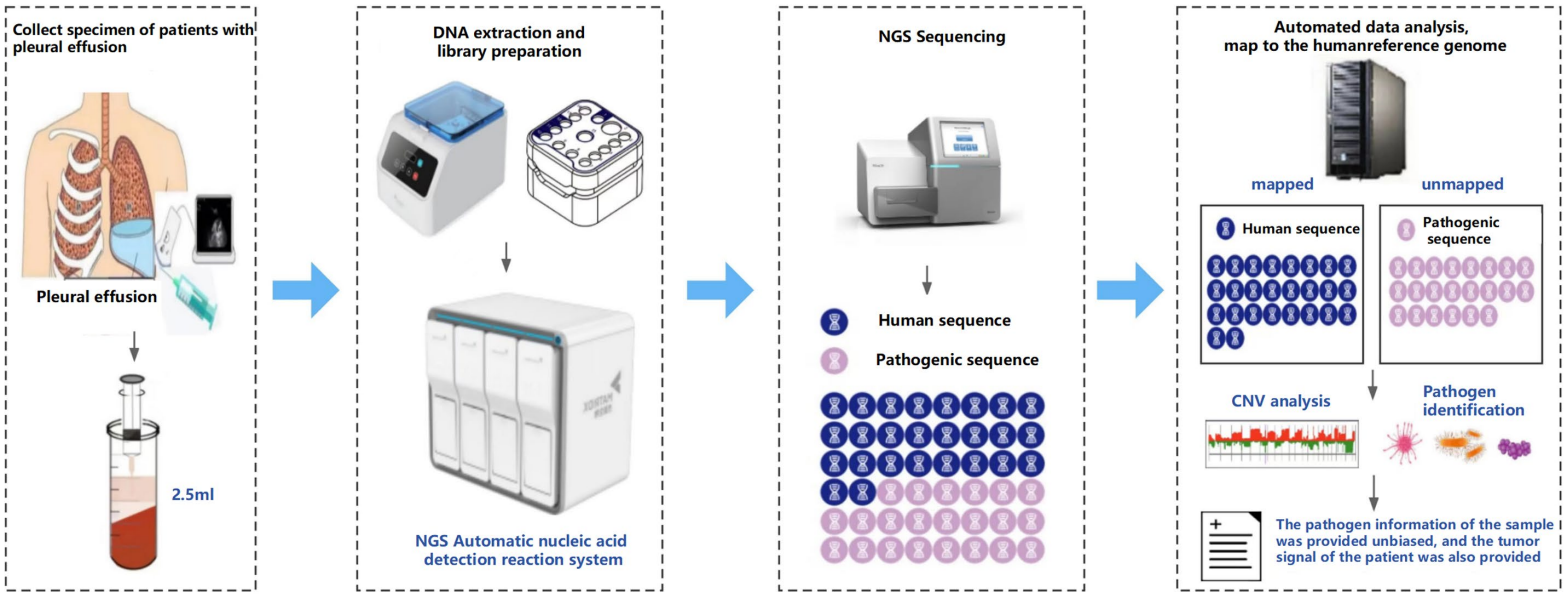
Workflow of onco-mNGS.

Quality control metrics were strictly maintained, with each sample yielding 10–20 million raw reads. For reliable CNV analysis, samples were required to meet the following quality thresholds: (1) a minimum of 1 million human host sequences and (2) a GC content ratio below 0.44. The comprehensive workflow of the mNGS procedure is schematically represented in Figure 2.

### 2.6 CNV and pathogen detection with mNGS data simultaneously

According to a previous study (19), sequencing reads were aligned with the human reference genome (hg19), with only unique, mapped reads selected for subsequent analysis. The reference genome was segmented into continuous windows of fixed length to determine the read depth of each window, which was then normalized to the total reads of each sample. The copy number ratio of each window was obtained by dividing the normalized read depth by the average read depth in the reference dataset. Afterwards, the fused least absolute shrinkage and selection operator (LASSO) method (a generalization of the LASSO penalty for sequential signal smoothing with sparsity) was applied to log2-transformed copy number ratios. Smoothed adjacent windows with similar ratios were merged into segments with chromosome positions and average ratios annotated. The copy number of each segment was calculated according to the average ratio and normal copy number of the corresponding chromosome and then compared with preset thresholds to validate the obtained CNV.

Then, the unmapped reads determined while aligning against the human genome were further used for pathogen detection as followed. Firstly, non-human reads were quickly classified using Kraken2 (20) by alignment against the NCBI reference sequence database. Then, the classified sequences were aligned against the microbial RefSeq database with bowtie2 (21) for verification. Next, BLAST (version 2.9.0+) alignment to the nucleotide database was conducted to validate candidate reads, for which Kraken2 and Bowtie2 data were inconsistent (22). Microbial reads identified from a library were reported if: (1) the sequencing data passed quality control filters (library concentration > 10 pM, Q20 > 85%, Q30 > 80%); (2) negative control (NC) in the same sequencing run does not contain the species or the reads per million reads (RPM)_sample_/RPM_NC_ ≥ 5, as a cutoff for discriminating true-positives from background contaminations (18). Finally, potential pathogens were selected from the results of the above analysis according to the clinical phenotype.

## 3 Results

### 3.1 Patient characteristics

During the study period from March 2022 to February 2023, 40 patients were initially screened, with 35 meeting the inclusion criteria and subsequently enrolled in this investigation. The demographic and clinical characteristics of the study cohort are comprehensively summarized in Table 1, while detailed individual patient data, including diagnostic outcomes and laboratory findings, are presented in Supplementary Table S1.

**Table 1:** Clinical characteristics of the patients enrolled in this study.

Characteristic *N* (%)		ALL (*N* = 35)	MPE (*N* = 10)	TPE (*N* = 8)	Others (*N* = 17)
Age	Median [range], y	70 [20–89]	65.5 [20–87]	63 [32–83]	75 [40–89]
Sex	Male	23 (65.7)	8 (80)	6 (75)	9 (52.9)
	Female	12 (34.3)	2 (20)	2 (25)	8 (47.1)
Symptom	Fever	7 (20)	1 (10)	2 (25)	4 (23.5)
	Cough, sputum	21 (60)	7 (70)	2 (25)	12 (70.6)
	Shortness of breath	19(54.3)	7 (70)	5 (62.5)	7 (41.2)
	Chest pain	12 (34.3)	2 (20)	4 (50)	6 (35.3)
	Fatigue	3 (8.6)	1 (10)	0 (0)	2 (11.8)
Underlying diseases^*^	Not reported	12 (34.3)	6 (60)	5 (62.5)	1 (5.9)
	COPD	3 (8.6)	1 (10)	0 (0)	2 (11.8)
	DM	7 (20)	2 (20)	0 (0)	5 (29.4)
	HBP	17 (48.6)	2 (20)	3 (30)	12 (70.6)
	Cerebrovascular disease	5 (14.3)	1 (10)	1 (12.5)	3 (17.6)
	Cardiovascular disease	6 (17.1)	0 (0)	2 (25)	4 (23.5)
	Liver disease	4 (11.4)	2 (20)	0 (0)	2 (11.8)
	Renal disease	4 (11.4)	0 (0)	2 (20)	2 (11.8)
History of malignancy		5 (14.3)	1 (10)	0 (0)	4 (23.5)

MPE, malignant pleural effusion; TPE, tuberculous pleural effusion; TB, tuberculosis; COPD, chronic obstructive pulmonary disease; HBP, hypertension; DM, diabetes mellitus.

* 18 patients (3 MPE, 3 TPE, and 12 other cases, who were non-TB infection and non-infection/malignant) had more than one underlying disease.

The cohort comprised predominantly male participants (65.7%, 23/35), with a median age of 70 years (range: 20–89 years). Comorbidities were prevalent, affecting 65.7% (23/35) of the study population. The most frequently observed conditions included hypertension (48.6%, 17/35), chronic obstructive pulmonary disease (COPD), diabetes mellitus (DM), hepatic disorders, renal impairment, cardiovascular disease, and cerebrovascular disease, reflecting the complex clinical profile of the patient population.

### 3.2 Etiology and pathogens of PE

**Figure 3:**
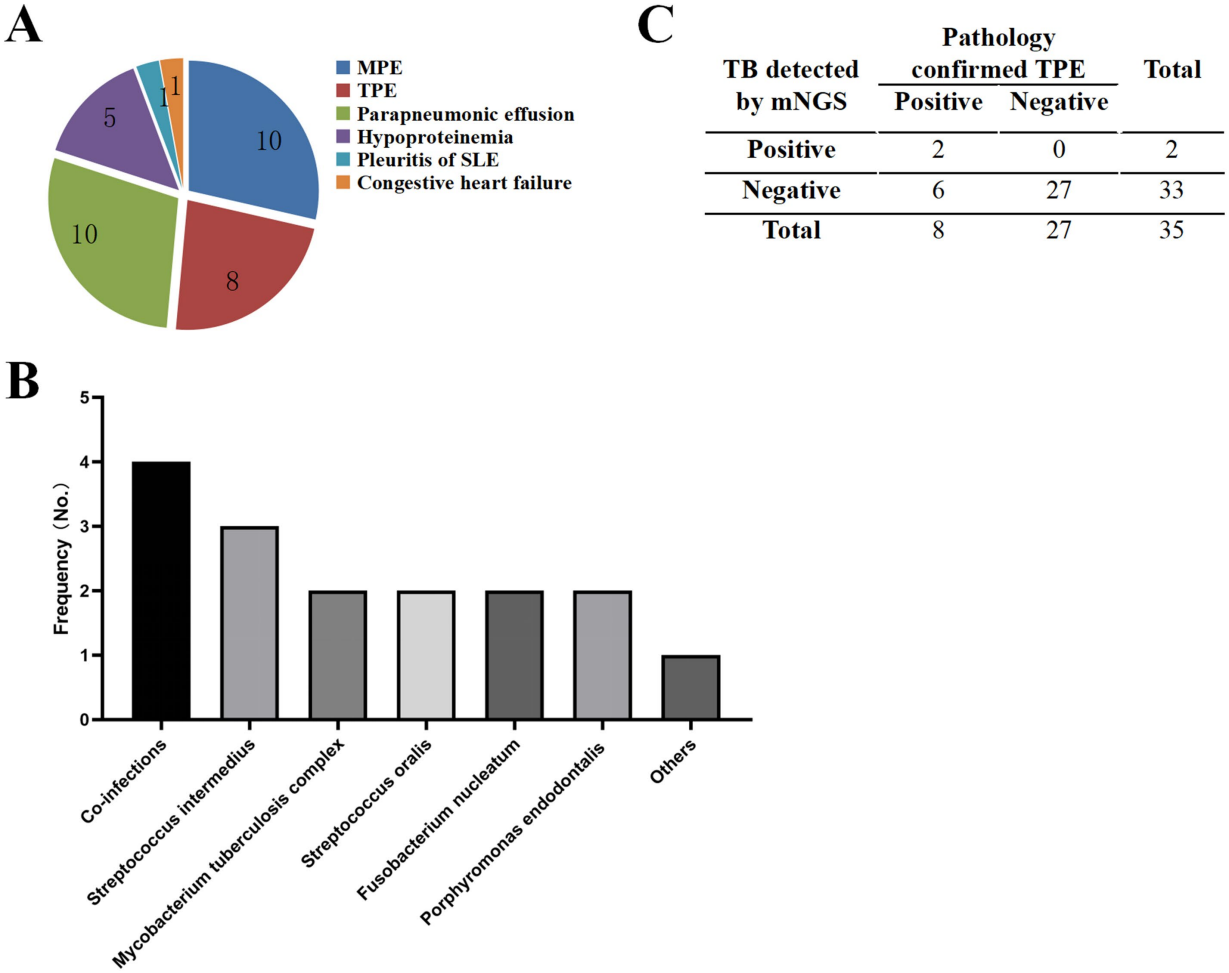
Characteristics of samples and pathogens detected by mNGS. **(A)** Distribution of pleural effusion types identified in the study cohort. **(B)** Microbial diversity detected by mNGS in 35 patients. “Others” category includes *Streptococcus pneumoniae*, *Escherichia coli*, *Pasteurella multocida*, *Lactobacillus crispatus*, *Serratia marcescens*, *Campylobacter rectus*, *Bacteroides fragilis*, *Bacteroides heparinolyticus*, and *Human polyomavirus type 1*. **(C)** Summary of mNGS results for TPE samples.

In this cohort, seven patients were diagnosed with non-infectious PE through comprehensive clinical evaluation, including detailed medical history review, systematic assessment of clinical manifestations, laboratory analyses, and follow-up monitoring. The etiological distribution included hypoproteinemia (*n* = 5), systemic lupus erythematosus (SLE)-associated pleuritis (*n* = 1), and congestive heart failure (*n* = 1), as illustrated in Figure 3A.

mNGS analysis identified infectious etiologies in 12 patients, revealing a diverse pathogen profile. The most prevalent pathogen was *Streptococcus intermedius* (12.5%, 3/24), followed by *Mycobacterium tuberculosis complex* (8.3%, 2/24), *Streptococcus oralis* (8.3%, 2/24), *Fusobacterium nucleatum* (8.3%, 2/24), and *Porphyromonas endodontalis* (8.3%,2/24). Notably, 16.6% (4/24) of cases demonstrated polymicrobial infections, as detailed in Figure 3B.

Among the eight patients meeting gold-standard diagnostic criteria for TPE, mNGS demonstrated a sensitivity of 25% (2/8) for *Mycobacterium tuberculosis* detection, while maintaining 100% specificity, as shown in Figure 3C.

### 3.3 mNGS for identifying MPE

**Figure 4:**
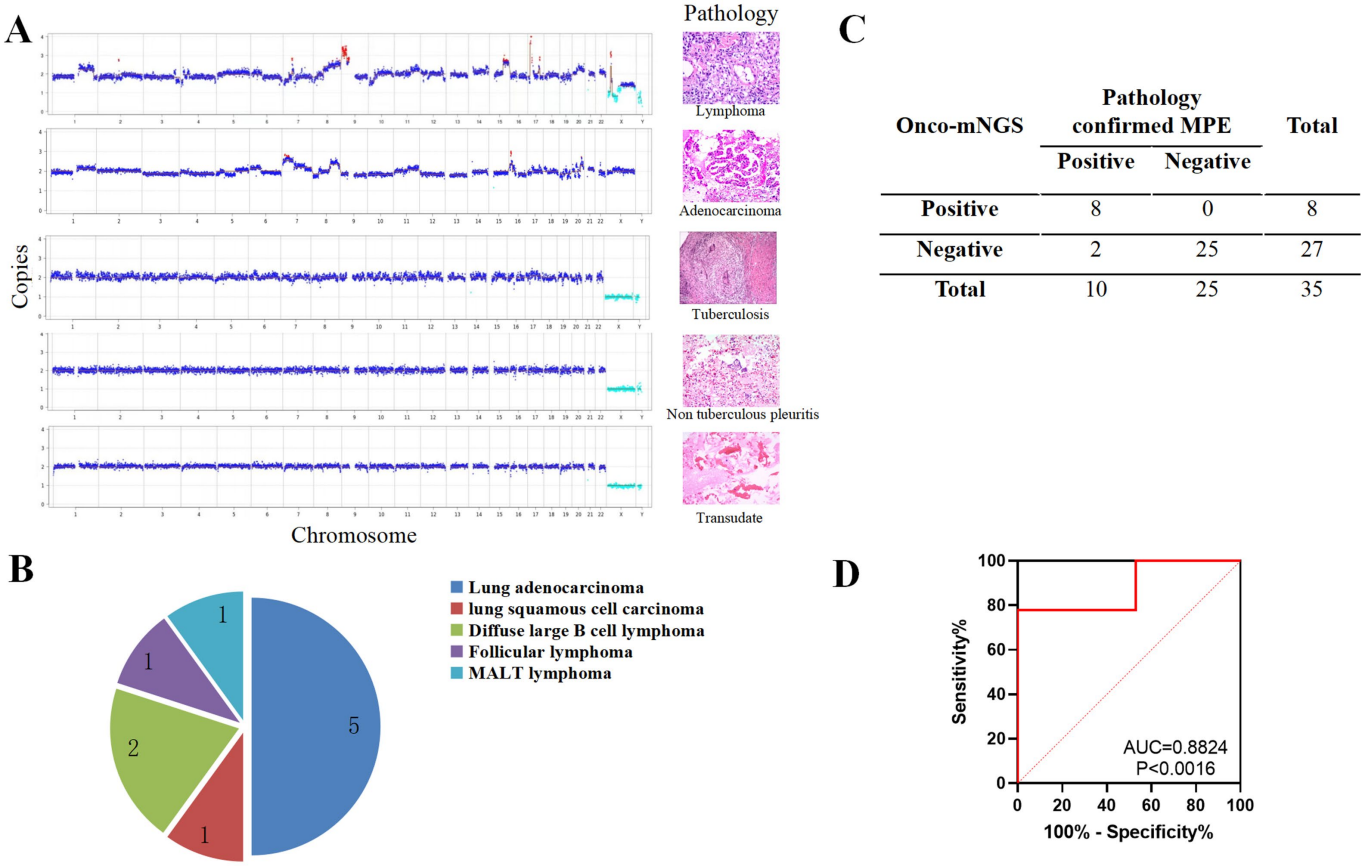
mNGS was used to perform a differential diagnosis of PE. **(A)** Comparison of CNV data derived from mNGS with histopathological findings in patients with PE. **(B)** Histopathological classification of MPE cases. **(C)** Summary of mNGS results for MPE cases. **(D)** Receiver operating characteristic (ROC) curve for CNV analysis. Red dots indicate copy numbers above 2.7 (amplification), light blue dots indicate copy numbers below 1.3 (deletion), and dark blue dots represent copy numbers between 1.3 and 2.7 (neutral).

Significant chromosomal disturbances were observed in the MPE group compared to the non-MPE group by mNGS (Figure 4A). Among the 35 patients, eight exhibited chromosomal abnormalities. Histopathological correlation revealed that five cases were pulmonary adenocarcinoma, two were diffuse large B-cell lymphoma, and one was follicular lymphoma (Figure 4B). Pathological examination confirmed malignancy in 10 patients, with lung adenocarcinoma being the most prevalent (50%, 5/10), followed by diffuse large B-cell lymphoma (20%, 2/10), follicular lymphoma, lung squamous cell carcinoma, and MALT lymphoma (10% each). The sensitivity and specificity of onco-mNGS for tumor detection were 80% (8/10) and 92.6% (25/27), respectively (Figure 4C). The diagnostic performance was further validated by an area under the receiver operating characteristic (ROC) curve (AUC) of 0.882 (Figure 4D).

## 4 Discussion

PE is frequently associated with malignant neoplasms or infectious diseases, particularly TB. However, distinguishing between these etiologies remains a significant clinical challenge. mNGS is an advanced diagnostic tool that enables comprehensive sequencing of all DNA within a sample, facilitating the identification of microorganisms and their genomic characteristics, such as antibiotic resistance genes (ARGs), virulence factors, and typing markers (23). This method allows for rapid (typically within 24 h) and unbiased detection of a broad spectrum of bacteria, viruses, and fungi by analyzing pathogen-derived nucleic acid fragments (24). Additionally, genomic instability, a hallmark of malignant neoplasms, has been extensively studied using whole-genome sequencing (15, 16, 25). In this study, we aimed to establish a minimally invasive, time-efficient, and comprehensive diagnostic approach using mNGS to simultaneously identify TPE and MPE.

Co-infections were identified in 16.6% (4/24) of patients, consistent with previous studies highlighting the prevalence of mixed pathogens in severe pneumonia, bacteremia, ocular infections, and central nervous system (CNS) infections (10, 26, 27). mNGS offers a rapid and precise method for detecting and characterizing diverse pathogens, which is particularly valuable for managing lung infections, especially in cases of polymicrobial infections (28). Notably, mNGS demonstrated a significantly higher detection rate for polymicrobial infections compared to traditional culture methods (70.97% vs. 12.90%, *p* < 0.001) (29). This technology provides a comprehensive genomic analysis of all microorganisms in a sample, including those that are unculturable, making it particularly advantageous for detecting mixed infections (30). However, mNGS cannot differentiate between live and dead pathogens, limiting its ability to distinguish colonization from active infection (28). Therefore, accurate interpretation of mNGS results requires integration with clinical data.

TB remains a major global health threat and the leading cause of death from a single infectious agent (31–33). Pleural tuberculosis (PT) is the most common extrapulmonary manifestation in adults (34, 35), with pleural involvement occurring in 3–5% of cases in non-endemic regions and up to 30% in endemic areas (32). TPE is typically characterized by exudative fluid with elevated adenosine deaminase (ADA) levels, lymphocytic predominance, and a straw-colored appearance, though mycobacterial culture yields are often low (36). Conventional diagnostic methods, such as Ziehl-Nielsen or Auramine staining, exhibit poor sensitivity (<10%) and cannot differentiate specific mycobacterial strains (7, 36). While Mtb culture remains the gold standard, its utility is limited by prolonged turnaround times (4–8 weeks) and low sensitivity (20–40%) in pleural effusion samples (7, 35–37). Emerging diagnostic approaches, such as interferon-γand interleukin-27 (IL-27) detection, face challenges related to cost, assay standardization, and accessibility in high-prevalence regions (35, 38). Although pleural tissue biopsy offers high accuracy, its invasiveness and associated risks (e.g., pain, bleeding, subcutaneous emphysema) limit its applicability (35). Recent studies have demonstrated the utility of mNGS for diagnosing pulmonary TB in sputum and BALF samples, with sensitivities ranging from 60 to 70% (39, 40). However, in our study, mNGS exhibited a sensitivity of only 25% (2/8) and a specificity of 100% for TPE detection, highlighting its limitations in this context. Potential reasons for this low sensitivity include: (1) the paucibacillary nature of TPE, as *Mtb* cultures are often negative, and PT is considered a delayed hypersensitivity response to *Mtb* or its metabolites (41, 42); (2) the intracellular growth characteristics of *Mtb*, which limit the release of extracellular nucleic acids (43); (3) the low mycobacterial yield in exudative pleural fluid, further complicating mNGS-based diagnosis.

PE specimens are often underutilized in clinical practice, and the potential of mNGS-based CNV analysis for cancer diagnosis remains underexplored. This study evaluated a novel strategy for simultaneous pathogen detection and malignancy prediction using a single mNGS assay, offering a minimally invasive alternative for patients who cannot tolerate or are at high risk for biopsy. The sensitivity and specificity of mNGS for tumor detection were 80% (8/10) and 92.6% (25/27), respectively. These findings align with previous studies demonstrating the utility of CNV analysis in various body fluids, including BALF and peritoneal fluid, with a sensitivity of 68% for cancer detection in conventionally negative cases (16). Additionally, mNGS has shown promise in diagnosing central nervous system malignancies in cerebrospinal fluid, with a sensitivity of 75% and specificity of 100% (44). Collectively, these studies underscore the potential of PE mNGS as a diagnostic tool for malignant neoplasms.

MPE is a common complication of metastatic disease, occurring in 15% of cancer patients (6, 45). It is most frequently associated with lung cancer (LC), followed by breast cancer (BC), lymphoma, gynecological cancers, and malignant mesothelioma (46). Adenocarcinoma accounts for 70–77% of MPE cases (47), consistent with our findings where lung adenocarcinoma was the predominant etiology. Lymphomas, particularly non-Hodgkin lymphomas (NHL), represent another significant cause of MPE, with diffuse large B-cell lymphoma (DLBCL) and follicular lymphoma being the most common subtypes (48). MPE occurs in 16–20% of NHL patients and is present in 10–30% of Hodgkin lymphoma (HL) cases at diagnosis, increasing to 60% during disease progression (49). Traditional diagnostic methods for lymphoma-associated MPE are often hindered by the scarcity of malignant cells in effusion samples (50). Our study identified lymphoma as a notable etiology of MPE, with DLBCL and follicular lymphoma cases detected. PE mNGS may emerge as a valuable tool for diagnosing lymphoma-related MPE in the future.

This study has limitations, including its small sample size. The pathogenesis of PT involves multiple factors, such as direct *Mtb* infection and pleural inflammation induced by *Mtb* metabolites. Huang et al. reported a sensitivity of 46.67% for mNGS in detecting PT, with high specificity (100%) and positive predictive value (100%) (51), aligning with our findings. Future large-cohort studies are needed to validate the clinical utility of mNGS for TPE diagnosis.

## 5 Conclusion

This study demonstrates the potential of mNGS for diagnosing MPE using PE specimens, with promising diagnostic performance. However, its utility for detecting *Mtb* in PE samples remains limited. These findings highlight mNGS as a promising minimally invasive tool for cancer diagnosis, though further validation in larger cohorts is warranted.

## Data Availability

The original contributions presented in the study are included in the article/Supplementary material, further inquiries can be directed to the corresponding authors.

## References

[1] Feller-Kopman D Light R. Pleural disease. N Engl J Med. (2018) 378:740–51. doi: 10.1056/NEJMra1403503, PMID: 29466146

[2] Jany B Welte T. Pleural effusion in adults-etiology, diagnosis, and treatment. Dtsch Arztebl Int. (2019) 116:377–86. doi: 10.3238/arztebl.2019.0377, PMID: 31315808 PMC6647819

[3] Light RW Macgregor MI Luchsinger PC Ball WC Jr. Pleural effusions: the diagnostic separation of transudates and exudates. Ann Intern Med. (1972) 77:507–13. doi: 10.7326/0003-4819-77-4-507, PMID: 4642731

[4] Cai Y Wang Y Shi C Dai Y Li F Xu Y. Single-cell immune profiling reveals functional diversity of T cells in tuberculous pleural effusion. J Exp Med. (2022) 219:1777. doi: 10.1084/jem.20211777, PMID: 35061012 PMC8789099

[5] Huang ZY Shao MM Zhang JC Yi FS du J Zhou Q. Single-cell analysis of diverse immune phenotypes in malignant pleural effusion. Nat Commun. (2021) 12:6690. doi: 10.1038/s41467-021-27026-9, PMID: 34795282 PMC8602344

[6] Feller-Kopman DJ Reddy CB DeCamp MM Diekemper RL Gould MK Henry T. Management of Malignant Pleural Effusions. An official ATS/STS/STR clinical practice guideline. Am J Respir Crit Care Med. (2018) 198:839–49. doi: 10.1164/rccm.201807-1415ST, PMID: 30272503

[7] Gopi A Madhavan SM Sharma SK Sahn SA. Diagnosis and treatment of tuberculous pleural effusion in 2006. Chest. (2007) 131:880–9. doi: 10.1378/chest.06-2063, PMID: 17356108

[8] Luo P Mao K Xu J Wu F Wang X Wang S. Metabolic characteristics of large and small extracellular vesicles from pleural effusion reveal biomarker candidates for the diagnosis of tuberculosis and malignancy. J Extracell Vesicles. (2020) 9:1790158. doi: 10.1080/20013078.2020.1790158, PMID: 32944177 PMC7480510

[9] Porcel JM. Biomarkers in the diagnosis of pleural diseases: a 2018 update. Ther Adv Respir Dis. (2018) 12:1753466618808660. doi: 10.1177/1753466618808660, PMID: 30354850 PMC6204620

[10] Blauwkamp TA Thair S Rosen MJ Blair L Lindner MS Vilfan ID. Analytical and clinical validation of a microbial cell-free DNA sequencing test for infectious disease. Nat Microbiol. (2019) 4:663–74. doi: 10.1038/s41564-018-0349-6, PMID: 30742071

[11] Chen H Yin Y Gao H Guo Y Dong Z Wang X. Clinical utility of in-house metagenomic next-generation sequencing for the diagnosis of lower respiratory tract infections and analysis of the host immune response. Clin Infect Dis. (2020) 71:S416–26. doi: 10.1093/cid/ciaa1516, PMID: 33367583

[12] Hu Y Kang Y Liu X Cheng M Dong J Sun L. Distinct lung microbial community states in patients with pulmonary tuberculosis. Sci China Life Sci. (2020) 63:1522–33. doi: 10.1007/s11427-019-1614-0, PMID: 32303963

[13] Chernow B Sahn SA. Carcinomatous involvement of the pleura: an analysis of 96 patients. Am J Med. (1977) 63:695–702. doi: 10.1016/0002-9343(77)90154-1, PMID: 930945

[14] Zamboni MM da Silva CT Jr Baretta R Cunha ET Cardoso GP. Important prognostic factors for survival in patients with malignant pleural effusion. BMC Pulm Med. (2015) 15:29. doi: 10.1186/s12890-015-0025-z, PMID: 25887349 PMC4379612

[15] Hanahan D Weinberg RA. Hallmarks of cancer: the next generation. Cell. (2011) 144:646–74. doi: 10.1016/j.cell.2011.02.013, PMID: 21376230

[16] Gu W Talevich E Hsu E Qi Z Urisman A Federman S. Detection of cryptogenic malignancies from metagenomic whole genome sequencing of body fluids. Genome Med. (2021) 13:98. doi: 10.1186/s13073-021-00912-z, PMID: 34074327 PMC8167833

[17] Guo Y Li H Chen H Li Z Ding W Wang J. Metagenomic next-generation sequencing to identify pathogens and cancer in lung biopsy tissue. EBioMedicine. (2021) 73:103639. doi: 10.1016/j.ebiom.2021.103639, PMID: 34700283 PMC8554462

[18] Luan Y Hu H Liu C Chen B Liu X Xu Y. A proof-of-concept study of an automated solution for clinical metagenomic next-generation sequencing. J Appl Microbiol. (2021) 131:1007–16. doi: 10.1111/jam.15003, PMID: 33440055

[19] Su J Han X Xu X Ding W Li M Wang W. Simultaneous detection of pathogens and tumors in patients with suspected infections by next-generation sequencing. Front Cell Infect Microbiol. (2022) 12:892087. doi: 10.3389/fcimb.2022.892087, PMID: 35755839 PMC9218804

[20] Wood DE Lu J Langmead B. Improved metagenomic analysis with kraken 2. Genome Biol. (2019) 20:257. doi: 10.1186/s13059-019-1891-0, PMID: 31779668 PMC6883579

[21] Langmead B Salzberg SL. Fast gapped-read alignment with bowtie 2. Nat Methods. (2012) 9:357–9. doi: 10.1038/nmeth.1923, PMID: 22388286 PMC3322381

[22] Zhang D Zhang J du J Zhou Y Wu P Liu Z. Optimized sequencing adaptors enable rapid and real-time metagenomic identification of pathogens during runtime of sequencing. Clin Chem. (2022) 68:826–36. doi: 10.1093/clinchem/hvac024, PMID: 35290433

[23] Heitz M Levrat A Lazarevic V Barraud O Bland S Santiago-Allexant E. Metagenomics for the microbiological diagnosis of hospital-acquired pneumonia and ventilator-associated pneumonia (HAP/VAP) in intensive care unit (ICU): a proof-of-concept study. Respir Res. (2023) 24:285. doi: 10.1186/s12931-023-02597-x, PMID: 37968636 PMC10648381

[24] Chiu CY Miller SA. Clinical metagenomics. Nat Rev Genet. (2019) 20:341–55. doi: 10.1038/s41576-019-0113-7, PMID: 30918369 PMC6858796

[25] Nagahashi M Shimada Y Ichikawa H Kameyama H Takabe K Okuda S. Next generation sequencing-based gene panel tests for the management of solid tumors. Cancer Sci. (2019) 110:6–15. doi: 10.1111/cas.13837, PMID: 30338623 PMC6317963

[26] Li Y Deng X Hu F Wang J Liu Y Huang H. Metagenomic analysis identified co-infection with human rhinovirus C and bocavirus 1 in an adult suffering from severe pneumonia. J Infect. (2018) 76:311–3. doi: 10.1016/j.jinf.2017.10.012, PMID: 29111306 PMC7126302

[27] Wilson MR Sample HA Zorn KC Arevalo S Yu G Neuhaus J. Clinical metagenomic sequencing for diagnosis of meningitis and encephalitis. N Engl J Med. (2019) 380:2327–40. doi: 10.1056/NEJMoa1803396, PMID: 31189036 PMC6764751

[28] Zhao Y Zhang W Zhang X. Application of metagenomic next-generation sequencing in the diagnosis of infectious diseases. Front Cell Infect Microbiol. (2024) 14:1458316. doi: 10.3389/fcimb.2024.1458316, PMID: 39619659 PMC11604630

[29] Wang C Yin X Ma W Zhao L Wu X Ma N. Clinical application of bronchoalveolar lavage fluid metagenomics next-generation sequencing in cancer patients with severe pneumonia. Respir Res. (2024) 25:68. doi: 10.1186/s12931-023-02654-5, PMID: 38317206 PMC10840150

[30] Wooley JC Godzik A Friedberg I. A primer on metagenomics. PLoS Comput Biol. (2010) 6:e1000667. doi: 10.1371/journal.pcbi.1000667, PMID: 20195499 PMC2829047

[31] Bagcchi S. WHO's global tuberculosis report 2022. Lancet Microbe. (2023) 4:e20:e20. doi: 10.1016/S2666-5247(22)00359-7, PMID: 36521512

[32] Chakaya J Petersen E Nantanda R Mungai BN Migliori GB Amanullah F. The WHO Global Tuberculosis 2021 Report - not so good news and turning the tide back to End TB. Int J Infect Dis. (2022) 124:S26–9. doi: 10.1016/j.ijid.2022.03.011, PMID: 35321845 PMC8934249

[33] Reid MJA Arinaminpathy N Bloom A Bloom BR Boehme C Chaisson R. Building a tuberculosis-free world: the lancet commission on tuberculosis. Lancet. (2019) 393:1331–84. doi: 10.1016/S0140-6736(19)30024-8, PMID: 30904263

[34] Li T Yan X du X Huang F Wang N Ni N. Extrapulmonary tuberculosis in China: a national survey. Int J Infect Dis. (2023) 128:69–77. doi: 10.1016/j.ijid.2022.12.005, PMID: 36509333

[35] Lo Cascio CM Kaul V Dhooria S Agrawal A Chaddha U. Diagnosis of tuberculous pleural effusions: a review. Respir Med. (2021) 188:106607. doi: 10.1016/j.rmed.2021.106607, PMID: 34536698

[36] Shaw JA Irusen EM Diacon AH Koegelenberg CF. Pleural tuberculosis: A concise clinical review. Clin Respir J. (2018) 12:1779–86. doi: 10.1111/crj.12900, PMID: 29660258

[37] Light RW. Update on tuberculous pleural effusion. Respirology. (2010) 15:451–8. doi: 10.1111/j.1440-1843.2010.01723.x, PMID: 20345583

[38] Mollo B Jouveshomme S Philippart F Pilmis B. Biological markers in the diagnosis of tuberculous pleural effusion. Ann Biol Clin (Paris). (2017) 75:19–27. doi: 10.1684/abc.2016.1201, PMID: 28057604

[39] Jin X Li J Shao M Lv X Ji N Zhu Y. Improving suspected pulmonary infection diagnosis by Bronchoalveolar lavage fluid metagenomic next-generation sequencing: a multicenter retrospective study. Microbiol Spectr. (2022) 10:e0247321. doi: 10.1128/spectrum.02473-21, PMID: 35943274 PMC9431624

[40] Liu X Chen Y Ouyang H Liu J Luo X Huang Y. Tuberculosis diagnosis by metagenomic next-generation sequencing on Bronchoalveolar lavage fluid: a cross-sectional analysis. Int J Infect Dis. (2021) 104:50–7. doi: 10.1016/j.ijid.2020.12.063, PMID: 33359946

[41] Aktas E Ciftci F Bilgic S Sezer O Bozkanat E Deniz O. Peripheral immune response in pulmonary tuberculosis. Scand J Immunol. (2009) 70:300–8. doi: 10.1111/j.1365-3083.2009.02294.x, PMID: 19703020

[42] Porcel JM. Tuberculous pleural effusion. Lung. (2009) 187:263–70. doi: 10.1007/s00408-009-9165-3, PMID: 19672657

[43] Zhou X Wu H Ruan Q Jiang N Chen X Shen Y. Clinical evaluation of diagnosis efficacy of active Mycobacterium tuberculosis complex infection via metagenomic next-generation sequencing of direct clinical samples. Front Cell Infect Microbiol. (2019) 9:351. doi: 10.3389/fcimb.2019.00351, PMID: 31681628 PMC6813183

[44] Gu W Rauschecker AM Hsu E Zorn KC Sucu Y Federman S. Detection of neoplasms by metagenomic next-generation sequencing of cerebrospinal fluid. JAMA Neurol. (2021) 78:1355–66. doi: 10.1001/jamaneurol.2021.3088, PMID: 34515766 PMC8438621

[45] Bibby AC Dorn P Psallidas I Porcel JM Janssen J Froudarakis M. ERS/EACTS statement on the management of malignant pleural effusions. Eur J Cardiothorac Surg. (2019) 55:116–32. doi: 10.1093/ejcts/ezy258, PMID: 30060030

[46] Penz E Watt KN Hergott CA Rahman NM Psallidas I. Management of malignant pleural effusion: challenges and solutions. Cancer Manag Res. (2017) 9:229–41. doi: 10.2147/CMAR.S95663, PMID: 28694705 PMC5491570

[47] Lepus CM Vivero M. Updates in effusion cytology. Surg Pathol Clin. (2018) 11:523–44. doi: 10.1016/j.path.2018.05.003, PMID: 30190139

[48] Skok K Hladnik G Grm A Crnjac A. Malignant pleural effusion and its current management: a review. Medicina (Kaunas). (2019) 55:490. doi: 10.3390/medicina55080490, PMID: 31443309 PMC6723530

[49] Wang Z Wu Y‑B Xu L‑L Jin M‑L Diao X‑L Wang X‑J. Diagnostic value of medical thoracoscopy in malignant pleural effusion induced by non-Hodgkin's lymphoma. Oncol Lett. (2017) 14:8092–9. doi: 10.3892/ol.2017.7226, PMID: 29344253 PMC5755161

[50] Alexandrakis MG Passam FH Kyriakou DS Bouros D. Pleural effusions in hematologic malignancies. Chest. (2004) 125:1546–55. doi: 10.1378/chest.125.4.1546, PMID: 15078773

[51] Huang F Wang H Qiao R Peng Q Zhao C Miao L. Diagnostic accuracy and microbial profiles of tuberculous pleurisy: a comparative study of metagenomic next generation sequencing and Gene Xpert *Mycobacterium tuberculosis*. Front Cell Infect Microbiol. (2023) 13:e20. doi: 10.3389/fcimb.2023.1243441, PMID: 38089819 PMC10711093

